# A ground-up “Quaternary” innovation strategy for South Korea using entrepreneurial ecosystem platforms

**DOI:** 10.1186/s40852-017-0061-4

**Published:** 2017-07-12

**Authors:** Philip Cooke

**Affiliations:** Mohn Center for Innovation and Regional Development, West Norway University of Applied Sciences, Bergen, Norway

**Keywords:** South Korea, Quaternary sector, Deindustrialisation, Chaebol, Crossover innovation, Thin globalisation, High profitability

## Abstract

This paper offers an account of the recent economic slowdown in the growth trajectory formerly enjoyed by South Korea as one of the first “Asian Tigers”. Indicators are provided that, unlike the others, Hong Kong, Singapore and Taiwan that have continued their upward profile, South Korea has stagnated. It is argued that the others and some more recent Asian growth economies have moved upwards to higher value, high skill and high profitability levels and deindustrialising as they did so. This even applies to recent breakthrough economies like China and Vietnam. In each case, “financialization” has been an important element in the growth of the Quaternary economy, even in such relative newcomers as Vietnam, where privatization of services has attracted private equity and other foreign direct investment financiers. Thus manufacturing is less pronounced than it was. Meanwhile, South Korea has a weak international presence of banks and other financial sectors because of the domestic focus in its indigenous growth model. Other weaknesses of closed versus open innovation and “cronyism” at the behest of the Chaebol system can be laid at the door of South Korea’s traditional conglomerates. A different model of “thin globalisation” led by knowledge-intensive high-tech, biotech and cleantech with prodigious financialization is characteristic of the new fast-growth regions and countries elsewhere, notably Israel, Silicon Valley and Cambridge. Here flattened hierarchies, reliable networking, and “crossover” innovation are pronounced and from which South Korean industrialists and policymakers could usefully learn to recover past growth performance.

## Introduction

The reason for this paper is that South Korea’s economy has stagnated in the secondary and tertiary developmental paradigms, similar to Japan, while the other “Asian Tigers” have forged ahead into the “Quaternary”. In this contribution, the plan is to reconsider the importance of “embeddedness” to regional economic development. One reason for this is that recent research in economic sociology has raised questions about its contemporary usefulness given critique of two elements: one intrinsic to the perspective; the other being a feature of evolutionary tendencies in the political economy of today and the near future. In brief, the critique draws attention to a recent questioning of “embeddedness” thinking treating the “economy” and specifically the “market” as an asocial constraint that limits social action on, for example, employment and the need for human labour (Ford, 2015). Thus social action is precisely what embeddedness refers to as the social fabric within which all social action is imbricated in societal relations, including economic ones. The second question refers to an emergent characteristic of contemporary political economy that is almost the reverse: namely in a political economy which has become increasingly “financialized” (Krippner, 2011) in which automated trading systems, electronic matching engines, varieties of decision algorithm and artificial intelligence (AI) have grown, how feasible is it for individuals to be socially embedded in what appears to be an increasingly “postsocial” economic world? In other words, has the market become “performative” (MacKenzie, 2008) in mimicking the theorems of neoclassical economics and can social action still control techniques that have developed an effectively asocial way of functioning?

Second, in re-thinking industrial policy when “normal market processes” reach hitherto unhindered obstacles, it may be helpful to treat it as a discovery process. So the appearance of economic obstacles reveals that government has imperfect information. This may further hinder its capability to overcome problems associated with inappropriate innovation to assist removal of such obstacles. Accordingly, as the handmaiden of policy, the result will express government failure. But since interventions are meant to smooth the flow of markets by means of market correcting initiative, absence of accomplishment is also suffered as “market failure” by the private sector. In some quarters, entrepreneurial demand for such innovation is low because private actors perceive new activities to be of low profitability (Rodrik, 2004).

However, thirdly, if through the lens of “financialization”, we look at certain micro-economies paying attention also to macroeconomic level data, we see profitability is high for what we refer to (after Rodrik, 2011) as “The Quaternary,” we see low profitability lies in manufacturing, services and – worst of all – agriculture. But productivity comes from selling Quaternaries to them. Thus it is innovation that enables restructuring and productivity growth, which are often constrained, to repeat, not on the supply side but on the demand side. So the developmental dilemma is that innovation is often undercut by lack of demand from its potential users in the real economy – the entrepreneurs. So, while government needs to evolve demand-inducing policies it may need to maintain its “embedded” autonomy from private interests. But it can elicit useful information from the “embedded” private sector by engagement with it. Such “embedded autonomy” (Evans, 1995) from the ground-up, may release demand impulses that help overcome developmental blockages, which is the aim of the following discussion.

## South Korea as an exemplar

South Korea experienced one of the most impressive turnarounds from being one of the lowest to highest GDP reference points after the Korean war (1950–1953), decades of imperialist exploitation by Japan (1910–1945) and an unreformed property rights land reform unaffected by centuries of feudalistic social relations. Growth was successfully achieved by interventionist government land reform, industry policies and the state as “global controller” institution for national economic organisation of the *Chaebols* developing a growth sector focus, especially in heavy engineering and light manufacturing. Thus large-scale corporate and government “embeddedness” expressed a good record on public administration of, for example, “export subsidies” by South Korea (Rodrik, 2011). For a time government policy on heavy investment in fossil fuel and exceptional reliance on nuclear energy contributed to rapid post-war growth.

But more recently, a hitherto prevailing “anti-green” policy perspective persisted in South Korea while elsewhere pollution and sustainability concerns were already being addressed in other OECD economies. Suddenly attention was paid regarding South Korea’s “fossil & nuclear” legacy energy policy when change occurred by Presidential Decree in 2010. This was recently re-prioritised as a leading intervention by new president, Moon Jae-In in 2017. It can be stated that South Korea retained its “embedded autonomy” leadership profile but that it had become outdated. Latterly, some of the leading developmental large firms have revealed problems, e.g. Hanjin, bankrupted in 2017, Samsung (SDI – “closed innovation and battery fires” internal supplier) and evidence of “cronyism” and corruption. Thus the hitherto harmonious implementation of “investment guarantees” had begotten the problem of “cronyism”. This took the form of a presidential indictment in relation to a $38 billion “transfer” from Samsung. Subsequently, evidence of expensive gifts to former President Park from Lotte Inc. for $17million and smaller cosmetic surgery infractions involving 17 Blue House visits were added to the indictment. So the model of post-war economic growth became shaky shortly after the local variant of the killer disease SARS (MEIRS) was also found to have origins in South Korea’s leading Samsung medical clinic.

**Fig. 1:**
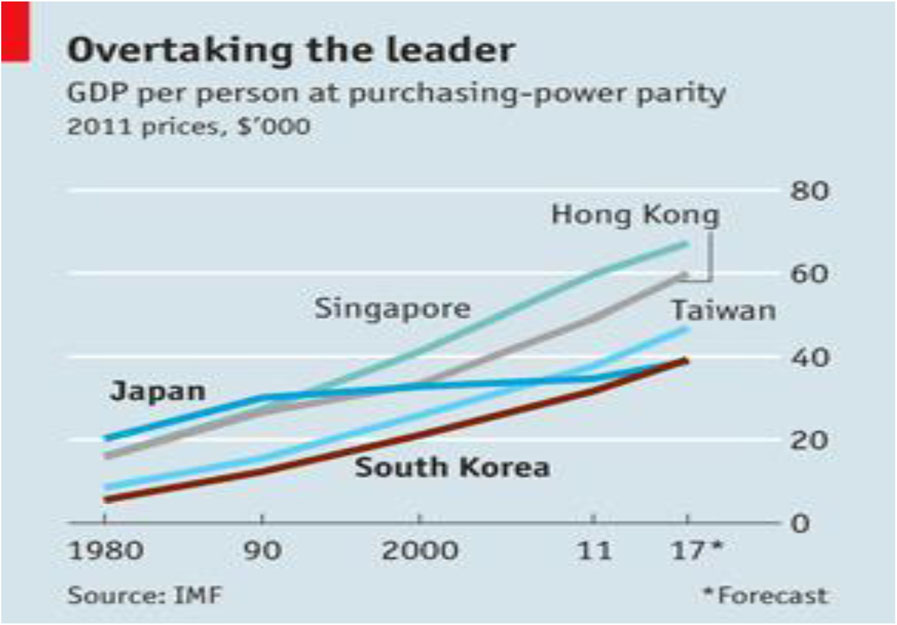
Overall comparative GDP in selected Asian economies 1980–2017. *Source*: (IMF, 2011)

However, because of its emphasis on “imitating” the Japanese “developmental state” model of rapid industrialisation by major investment in heavy industry, notably steel and shipbuilding, followed by light engineering in consumer goods (automotives and electronics) South Korea experienced a somewhat asymmetrical “financialization” if indeed that is a correct descriptor. Because the *Chaebols* contained their own banks, each supplying preferential investment to its industrial “family” South Korea never developed the kind of international banking system that other “Asian Tigers”, notably Hong Kong and Singapore did at the centre of *their* developmental strategies. This was for good reasons given that those two island economies had already developed as trade, commerce and financial centres before independence and they had little option but to do so, although both later developed profitable “quaternary” economic activities like ICT and biotechnology to accompany their financialization. Significantly, South Korea’s GDP per person (purchasing-power parity; PPP) has long lagged somewhat compared to the three “Tiger Island” economies. But, it is also noteworthy that Japan’s relative economic stagnation since 1990 means that South Korea was expected (by IMF) to by-pass Japan in 2017 as did Singapore in 1993, Hong Kong in 1997 and Taiwan in 2010 (Fig. 1). But the most startling re-ranking will be when South Korea becomes richer than Japan, since in 1980 South Korea’s GDP per person was barely a quarter the level of Japan’s.

However, our key indicator for the future is the trajectory of the “Quaternary” measured by profitability. While the trend in profitability had been upward, 2013 saw a decline in bank profitability. South Korean banks reported $8.2bn in combined net profits in 2012, a drop of 23.2% from 2011, as their net interest margin – a key measure of banks’ profitability – fell to 2.1%, its lowest level of the past decade apart from the crisis years of 2007–9. The country’s banks face squeezing margins due to their heavy reliance on *interest* income, while their non-interest income remains small. To compensate, they are under increasing pressure to expand into non-banking activities like insurance (KB Financial Group) and derivatives (e.g. Woori Finance) rather unsuccessfully. South Korean banks remain weak in investment banking, which requires thorough risk control. Consumer credit offers little room for growth, given the already high levels of debt among Korean households. Also, penetrating foreign markets has been a difficult, due to their lack of brand value and international networks (Jung-a, 2013). Moreover, the profitability of the four main Seoul-based companies, Shinhan Financial Group Co. Ltd., Hana Financial Group Inc., KB Financial Group Inc. and Woori Bank dropped for two consecutive years from 2013. Their combined profit declined by half, to 4.78 trillion won (US$4 billion) at the end of 2013 from 9.19 trillion won (US$8 billion) 2 years earlier.

If we move to other knowledge activities, we find profitability down there too. South Korea’s 30 largest business groups saw their profitability plunge to the lowest level since the global financial crisis in 2008. The combined operating profit of the nation’s 30 largest conglomerates by assets came to 57.56 trillion won (US$49.34 billion) last year, down 4.3% from 60.17 trillion won (US$51.58 billion) in 2008. The figure decreased by as much as 34.8%, or 30.69 trillion won (US$26.31 billion), from the peak of 88.25 trillion won (US$75.65 billion) in 2010.

Their operating profits have rapidly decreased in the last 4 years from 82.39 trillion won (US$70.63 billion) in 2011, 76.16 trillion won (US$65.29 billion) in 2012, 70.4 trillion won (US$60.35 billion) in 2013, to 57.56 trillion won (US$49.34 billion) in 2014. Although the business profit rates of Samsung Group and Hyundai Motor Group were higher than those of 2008, the figures of both companies were showing a downward trend after hitting peaks in 2010 and 2011, respectively. Samsung’s operating profit rate reached 11% in 2010, then kept decreasing from 9.7% in 2012 and 8.9% in 2013 to 6.4% last year. For Hyundai Motor Group, the figure also dropped from 8.7% in 2011, 7.8% in 2012, and 7.2% in 2013 to 6.9% last year (Jung Suk-yee, 2015). HSBC, Europe’s biggest bank, further scaled down its South Korean operations by closing its retail business, following the sale of its insurance business in 2013. Thus outlook for consumer banking in South Korea remained a concern to firms there. Standard Chartered said earlier in 2013 that it had seen a decline in asset quality in the country and would reassess the value of its goodwill.

So in respect of the hypothesis of “financialization” - meaning the share of profits going to interest and dividends is growing, and the rate of profit considering nonfinancial corporates converges to the real rate of profitability - does not appear to be the case for South Korea. The reverse is the case, where the rate of financial outflow – that is, the sum of interest, dividends and rents relative to those of nonfinancial corporates – has remained relatively stable at 50% since the 1980s (Hart-Landsberg et al., 2017) Exporters are creating fewer jobs in South Korea as the *Chaebol* move production offshore to look for cheaper labour. That has left the domestic economy hurting: small and medium-sized businesses are still failing and the high-value services sector is lagging well behind other countries*.* According to the OECD: *“This has raised concerns about Korea’s traditional catch-up strategy led by exports produced by large Chaebol companies”,* the OECD Report on South Korea said in its recent study (OECD, 2016). There has also been increasing economic polarisation in the post 2008 downturn. Economic inequality increased noticeably during and after the 1997 crisis and the Great Recession of 2008–9. South Korea’s average Gini coefficient — a measure of inequality — for 1990–1995 was 0.258, but with rising inequality its coefficient increased to 0.298 in 1999. It continued to increase, reaching 0.315 in 2010. The same trend can be seen in income distribution: the share held by the top 10% of income holders divided by that of the bottom 10% has increased from 3.30 in 1990 to 4.90 in 2010. The income share of the top 1% was 16.6% of national income in 2012, not far short of the extremes in the US and much worse than in Japan (Roberts, 2017).

However, it is noteworthy that Singapore’s profitability has also recently been downgraded. Thus third quarter 2016 profitability of Singapore’s big three banks declined in asset quality because of their exposures to loans and investment in the oil and gas service companies. This goes against Rodrik’s (1995) revisionist but plausible explanation for the economic take-off of Singapore and Taiwan, which was the sharp increase in investment demand that took place in the early 1960s. The reason for this investment boom – unlike the accounts of such agencies as the IMF and World Bank that stress export orientation - is the efforts of the respective governments massively to enhance government co-ordination and fashioning of innovative measures to promote profitability. Profitability from export growth at the time was modest by comparison. A much more plausible explanation for the economic take-off is thus the sharp increase in investment demand that took place in the early 1960s. Rodrik’s (1995) heterodox argument is that in the early 1960s and thereafter the Korean and Taiwanese governments managed to engineer and enhance a significant increase in the private return to capital which increased profitability.

Space does not allow further analysis of these differences between the comparatively low “quaternary” economic development of the recent decade or two in South Korea’s growth trajectory. We simply assert from the limited evidence mobilised, that two important deductions can be made from the comparative data and analysis proposed by economic growth theorists such as Rodrik (1995, 2011) The first of these is that manufacturing employment and GDP share remain high in South Korea. Meanwhile some even later industrialising countries have already begun de-industrializing, notably China and Vietnam. Conversely, the “Tiger” economies, notably Singapore and Hong Kong, and their Asian successors have “financialized” their economies more than South Korea (e.g. also Vietnam) and have augmented such quaternary activity with other knowledge-intensive, high value, high skill and high profitability quaternary activities as in Taiwan. These include activities such as innovative ICT, software, systems design, medical biotechnology and R&D. In this respect their economic profiles are closer to those of regions like Silicon Valley, Greater Cambridgeshire and Israel, which are among the most knowledge-intensive “quaternary” regions in the world.

## Interactive or crossover innovation

In this section of the paper, we propose to utilise the information so far displayed alongside some key insights about the mechanisms that may help understanding of the differential trajectories of South Korea in comparison with some other Asian growth economies. We shall begin with outlining some key features of “innovation governance” in the advanced regional innovation systems listed at the end of the previous section before comparing and contrasting such governance with what has typified or diverged from that emergent new “innovation governance” mode. First, we may say that high-tech platform ecosystems or complexes like Silicon Valley, Cambridge and Israel do not display strong top-down governmental modes of economic decision-making in policy or strategy. In other words there is seldom, if ever, a peak committee in which economic deliberations that directly affect specific platform industries by producing detailed action-lines that favour or disfavour specific technologies. That is not to say that in a general way, certain bundles of “cross-cutting” new technology capabilities or problems that may indeed occur in the form of “technology pathologies” may be fashioned. These may evolve as broad frameworks for alerting or sensitising “actors of consequence” of a clearer priority of recognition by “policy champions”. A good example is “Homeland Security” which consists of many diverse but technologically interlocking targets, problems and opportunities. In the US as many as 17 different information and intelligence agencies engage directly with intelligence gathering at home and abroad. These involve mobilising “Big Data” gathering and analysis, algorithm writing, cybersecurity, cyberwarfare, including cyberforensics, drone design and applications and multiple kinds of tracking, verifying, intercepting and, if necessary, arresting or otherwise preventing “technology pathologies” from threatening individual lives and communities. Without labouring the point, such “crossover” innovation opportunities also occur, in different combinations but including overlaps across the boundaries of “Big Platforms” such as Biomedicine, Elderly Healthcare, Artificial Intelligence, Renewable Energy and Sustainable Mobility, sometimes “fuzzily” designed to meet “Societal Grand Challenges”.

Such often “post-political” activity bundles are moulded by “policy champions” of various kinds. For example, Artificial Intelligence, with its close linkages to Robotics and Nanotechnology has a few “protean” influential champions in the US such as Ray Kurzweil, an apologist for AI for decades (Ford, 2015; Barrat, 2013). Kurzweil himself is widely seen as an attention-seeking entrepreneur and proselytiser for only the positive implications of AI. He is influential, having his pedagogical efforts sponsored by, amongst other Californian businesses, Google, Genentech and Cisco Systems. His inventive effort has touched such technologies as optical character recognition, computer-generated speech and music synthesis, all of which relate to augmentation of human senses. He was awarded 20 doctoral degrees from the likes of Babson College, Bloomfield College, Clarkson University, DePaul University, Hofstra University, Michigan State University, Rensselaer Polytechnic Institute and Worcester Polytechnic Institute, and been honoured by US presidents Johnson, Reagan and Clinton. Among his awards to the technological, humanities and musical communities are the following: 2000 The Lemelson-MIT Prize. This $500,000 award is the largest in the U.S. in invention and innovation. 1999 The National Medal of Technology, the nation’s highest honor in technology. 1998 The Stevie Wonder / SAP “Vision Award” for Product of the Year a $150,000 prize (being used by the Kurzweil Foundation to provide scholarships to blind students), and the 2008 American Creativity Association Lifetime Achievement Award. It can obviously be agreed that the optimist Kurzweil is widely seen as a “crossover” innovator and an AI “champion” despite his cultist association with Silicon Valley’s “Singularity University” (reminiscent in some ways of L. Ron Hubbard and “Scientology”) which Kurzweil founded in 2008.

Without contemplating the “cultist” evangelizing of Kurzweil’s obsession with a fictitious fake version of the astrophysical phenomenon of the “singularity” when even light can no longer escape from a black hole in space, three things that follow are pertinent to our utilisation of his curriculum vita in support of the function of “champions” as arbiters of post-political action framing. First, it is noteworthy the extent to which Kurzweil’s innovative career expresses crossover innovativeness with respect to: the invention of a classical music synthesizing computer involving designing computer technologies such as machine reading to assist the disabled and to enrich the arts, including winning awards for film production. Second, the institutional nodes with which Kurzweil’s interaction occurs are solid entities in the worlds of academic research entrepreneurship, government and large corporations. After long advisory roles with firms listed above, he was in 2013 appointed head of engineering at Google. He had worked with Google’s co-founder Larry Page on special projects over several years. His executive appointment occurred as Google began assembling the largest artificial intelligence (AI) laboratory in existence. Acquisitions involved military robotics firm Boston Dynamics, thermostat maker Nest and cutting-edge Cambridge (UK) AI firm DeepMind. These were added to smaller purchases of Bot & Dolly, Meka Robotics, Holomni, Redwood Robotics and Schaft, and another AI startup, DNNresearch. It also hired Geoffrey Hinton, a British computer scientist who is rated the world’s leading expert on neural networks (Cadwallader, 2014). Finally, Kurzweil is an avid publicist for his serious and more questionable analyses and predictions having published seven books translated into 11 languages.

No other technology – specifically AI (with robotics [Ford, 2015] and nanotechnologies) – has anywhere near as “protean” the influence on key decision actors ranging from DARPA to Google as the aforementioned Ray Kurzweil but others take on relevant roles from other more sceptical viewpoints. Three of these, cited in Barrat (2013) include I. J. Good, Eliezer Yudkowski, and Stephen Omohundro. Good, who died at 92 in 2009, was a British expatriate mathematician and former Bletchley Park codebreaker colleague of Alan Turing. Good was responsible for coining the term “information explosion” to describe the impact of AI on human intelligence when it could be anticipated. Stanley Kubrick turned to Good as the adviser on the 1968 film 2001: A Space Odyssey. It was Jack Good with his insights on intelligent machines, who helped create the infamous character of HAL, the AI computer in the film. In Good’s seminal paper “Speculations concerning the first ultra-intelligent machine” he defined this – a forerunner to “Singularity” thinking - as follows:

Let an ultra-intelligent machine be defined as a machine that can far surpass all the intellectual activities of any man (sic) however clever. Since the design of machines is one of these intellectual activities, an ultra-intelligent machine could design even better machines; there would then unquestionably be an ‘intelligence explosion,’ and the intelligence of man would be left far behind. Thus the first ultra-intelligent machine is the *last* invention that man need ever make, provided that the machine is docile enough to tell us how to keep it under control. (Good, 1965)

Accordingly, Good was a “champion” and influential at the highest governmental, academic and corporate levels with crossover theoretical interests from Bayesian mathematics to computer programming design and manufacturing to film consultancy. Moreover, he was careful not to take an over-optimistic line on the controllability of AI unless - as he wrote – “docility” could be built into the resulting technology. Other more sceptical AI “champions” who take a more practical but still pessimistically inclined view regarding the difficulty of ensuring “docility” from future AI or “artificial general intelligence” (AGI) as they term it, include gurus such as Eliezer Yudkowski, and Stephen Omohundro, noted earlier and as profiled extensively in Barrat (2013). Omohundro is optimistic, but this is based on his underlying notion that all AI is lethal because of the well-known software engineering problem that much programming is bad work, i.e. sloppy and incompetent, as Microsoft Word users have known for decades for its almost constant de-bugging upgrades. Bad programming is estimated to cost the US economy $60 billion per year. This implies a vast need for “self-improving software” a variety of “evolutionary programming” that may evolve from currently practised “machine learning”. Article space disallows fuller explication of such potentially influential views, save to say that Yudkowski – who invented the AI Box – a kind of Turing machine that led some players of its “game” to believe that a “thinking engine” had been invented, insists AGI would be catastrophic for humanity unless it is designed to be “Friendly AI”, But as Barrat (2013) observes critics argue that progressing towards AGI is necessitated by the even greater dangers of “artificial specialised intelligence” (ASI) falling into the hands of:

“ so many reckless and dangerous nations on the planet – North Korea and Iran for example – and organised crime in Russia and state-sponsored criminals in China launching.....cyberattacks, relinquishment would simply cede the future to crackpots and gangsters” (Barrat, 2013, 200–01).

Hence we see the origins of the engineer’s linear determinist thinking enlarged prodigiously and apocalyptically. The initial “mindlessness” of contemporary incremental innovators is captured in the following statement from Uber founder and Chief Technical Officer (CTO) Oscar Salazar who admitted:

“We are adding technology to a society without thinking about the consequences. I think government, industry and society need to work more together, because it is going to get crazier and crazier.” (Fairchild, 2017).

Here – belatedly - is recognition that as governments fail adequately to regulate technological experiments, good champions are also hard to find when their infantile aspirations are mainly “disruptive” (Christensen, 1997) and informed by the likes of Facebook’s Mark Zuckerberg’s earlier mission statement to “move fast and break things” (the origin of bad programming; Taplin, 2017). It has finally dawned on the Ubernauts that, as Fairchild (2017) also notes:

“Advances in artificial intelligence and automation could mean as many as 50% of today’s US jobs will go away, according to some estimates. Joined on stage by other high-profile members of the tech community, (chair Kara) Swisher forced her panelists to defend Silicon Valley’s seeming incapability to take responsibility for the downstream effects of its innovation. (Ibid)

Most governments and tech entrepreneurs excuse their mindlessness regarding the effects of AI automation upon workforces by stressing the importance of retooling and reskilling the workforce for tech jobs in the future. As engineers, in the main, they completely fail to see the paradox that they are responsible for the future absence of positions that it will be futile to train anyone for (Streeck, 2016). We shall return to this conundrum of engineering’s linear model of non-reflective obtuseness later, but for the moment we cite Frey & Osborne’s (2013) estimate of 64 million US jobs (47% of the total) having the potential to be automated and thus disappear within “perhaps a decade or two” (Frey & Osborne, 2013).

## Policy without global controllers

This narrative demonstrates that technological policy innovation needs “Champions” although they do not have to be evangelical or cultist in their behaviour along the lines of Ray Kurzweil, even though he clearly fits in with a particular strand of American science fiction “envisioning” that suits the vacuous purposelessness of the careless engineering and software programming that clearly often characterises high-tech innovation processes. Even when there is some degree of “adult supervision” of highly sensitive explorative and purposeful algorithm design, other mistakes can be thoughtlessly committed. Thus the story of the “cyberecosystem” and its often dystopian as distinct from cyberutopian outcomes is often prefaced by reference to and discussion of the work of disaster sociologist Charles Perrow. In his oft-cited book “Normal Accidents” (Perrow, 1999) and in particular his critique of “tight coupling” describes a system whose parts have immediate and severe impact upon each other.

A case in point is the so-called “smart grid”, another is the financial system (MacKenzie, 2008) or food refrigeration system, healthcare system, defence system and so on. All of these and many other such systems are potentially vulnerable regarding “department of homeland security” (DHS) issues. In 2007 DHS tested the robustness of the grid at the Idaho National Laboratory by selecting a typical online turbine generator, “hacking” it and altering its settings. Accordingly, it malfunctioned as the turbine self-destructed from inside. Its “supervisory control and data acquisition” system (SCADA) the “global controller” encrypted security programming of the type used in many systems critical settings noted above failed. On the bass of this, a new kind of malicious software (malware) to cause such destruction to hostile systems was conceived. It was called Stuxware. It was specifically designed for the US (NSA) and Israeli intelligence services to destroy a Siemens logic controller used in a gas centrifuge nuclear fuel enrichment plant in Natanz, Iran by subverting its virus-prone MS Windows PC operating system. Spies carried flash drives releasing the Stuxnet virus throughout the plant’s local area networks (LAN) to identify undiscovered security holes in the operating system.

Stuxnet worked and, as noted, it was likely sponsored by US and Israeli intelligence agencies but the private Equation Group has been identified as advising in the US and Mossad’s agents activating the Israeli contribution. Un-named private consultants in Tel Aviv were also interviewed in website reports and testing is asserted to have been conducted at the Idaho National Laboratory nuclear research facility in the US (the same one where DHS conducted its stress-test) and at Dimona’s Negev Nuclear Research Center, Israel’s nuclear weapons facility. But, even so, Stuxnet, which was supposed to self-destruct after multiple malware operations, escaped, allowing thousands of copies to be accidentally distributed. Experts conclude that this fate is typical of the lack of thinking beyond the short-term that characterises “act fast and break things” thinking about the likelihood of “normal accidents” occurring. In 2017 variants of Stuxnet were implemented as WannaCry and (Not)Petya malware attacks by “ransomware” cyberfraudsters.

While this narrative does not seek to present platform ecosystems of the Quaternary kind under discussion as paragons of virtue, it is clear that even the tightest hierarchical control assumed to be typical of military hierarchies is not immune to major failures of administration predicted in Perrow’s (1999) “normal accidents” analysis. Although we have yet to turn to the implications of this narrative for the prodigious hierarchical control of *Chaebols* like Samsung in the South Korean context it should nevertheless give pause for concern during that national economy’s period of relative stagnation compared to the past. One reason is that a tradition of Neo-Confucianist hierarchy, obedience and control associated with *Chaebol* tradition is no longer the administrative power in industrial organization that it once was. More recently, notions of “flattened hierarchies”, “intrapreneurship” and “open innovation” have affected learning in some large corporate entities as they have struggled to compete with more flexible, nimble and agile regional and global supply and knowledge networks. Typically, this way of operating has characterised the SME platform ecosystems of the Quaternary activities pronounced in the Silicon Valley, Cambridge and Israeli set-ups and in the global financial “superhubs, “biomedical megacentres” that are nowadays the leading “frame” for learning innovative organizational “(non)governance” (Nadivi, 2017).

Accordingly a highly “networked” collaborative enterprise complex characterises, for example the “complex adaptive super-systems” that manage global financial systems. These elements of “superhub globalisation” with numerous nodes distributed across the planet contrast with the “thin globalisation” networks more typical of the other knowledge-intensive Quaternary activities associated with “Big Data”, cybersecurity, systems design, software algorithms, biopharmaceuticals and cleantech. As noted earlier, these have high rate of networking among technology entrepreneurs, university researchers and government or military representatives and clients. Such platform ecosystems are thus not top-down hierarchical administrative or bureaucratic systems in any meaningful way. It is thus a decentralised, associational “Innovation Advocacy” model of industry organization. There is usually no formal strategy; the main driver has been incremental, evolutionary, sometimes rapid, change. “Superhubs” for financial services and *biotech megacentres” particularly in the UK and US tend to be open not managed economies and there has been little or no recognisable industrial strategy The UK’s recent attempt at an industrial strategy in 2017 was disparagingly referred to as a political “toyshop” Finally, we now see, typically new innovation models emerging in the likes of Cambridge where “crossover” mutations from microelectronics to advanced combustion engines and healthcare are being fashioned (Eason & Dean, 2016).

## Cambridge innovation advocacy without a “global controller”

In Cambridge the “soft infrastructure” of entrepreneurship and innovation marketing support has the following intermediaries active at one or other time assisting the ICT, biotech, software and systems and cleantech sectors. The Cambridge Network, which links together members and provides services for academic entrepreneurship. St. John’s Innovation Centre incubation environment accelerated the growth of ambitious innovative start-up businesses. Cambridge Science Park, established in 1970 was the new setting on which the ICT cluster began to grow rapidly. There were some 39 new companies from 1960 to 1969. In the 1970s, 137 were formed. By 1990, there were 100 per year. These initiatives are now supported by knowledge-intensive intermediaries such as ideaSpace, which is a community of people in Cambridge starting high impact new ventures. Hence ideaSpace members are creating new business models (Kirk et al., 2016).

What we might term examples of “Soft Infrastructure, Soft Power” includes institutions like Cambridge Enterprise which helps Cambridge University students and academics to commercialise innovative ideas by establishing a business. In the field of biotechnology, One Nucleus is a not-for profit Biotechnology membership organisation which aims to maximise the global competitiveness of its members. Supporting this is Cambridge Biotechnology Campus, which houses 7000 professionals and scientists. Of significance also is The Wellcome Genome Campus is home to some of the world’s foremost institutes and organisations in genomics (Sanger Institute) and computational biology at Hinxton Tech Park. This facility is a long-established and highly valued support infrastructure also for biotechnology - Babraham Biosciences Incubator & Research Campus. A newer are a of cluster ecosystem evolution involves clean technology, represented by Cambridge CleanTech. This is also a member organisation for cleantech start-ups and evolved firms, replicating the “associational” or collaborative mode of start-up industry organisation. Bestriding this associational infrastructure are cluster-platform “champions”, notably Hermann Hauser, co-founder of Acorn with Christopher Curry, who was part of a *Cambridge II* initiative. Hauser’s venture capital company Amadeus (with funding from the likes of software transplant Microsoft) was a leading actor in helping start-up companies. Thereafter, in collaborative efforts to access support for growth in Cambridge, which by 2017 had grown to the status of a city-regional mayoralty consolidated as the Cambridge-Peterborough, built partly on the growth insights of the past partnership among Alec Broers (Vice-Chancellor of Cambridge University), spatial planner Marcial Echenique (Cambridge University School of Architecture and a transport planning specialist and David Cleevely (Analysys telecom consultant founder) who, collectively, decided to assist the – subsequently successful - attempt to develop Cambridge’s high-tech future.

This marked the evolution of “Cambridge Phenomenon 2”, (Segal et al., 2011) which in 1997–8 looked at various issues such as land use, transport systems and telephony. The aim was to seek to accommodate growth through new Science Park development to link the university and industry. The university saw need for seedcorn finance and participated in seed capital funds, including the Quantum Fund, and Cambridge Research and Innovation Ltd. Entrepreneurs also became venture capitalists: Amadeus Capital (Hermann Hauser); Merlin Ventures, a biotechnology fund (Chris Evans founded Chiroscience) and the Gateway Fund founded by local financier Nigel Brown. Thus the “champions” were able to envision how future growth rests on continued acquisition of research funding, understood as the key knowledge core of – especially – ICT and biotechnology innovation excellence. A future key is the identification of *flexible research funding* that furthers and fosters “knowledge at interfaces” (“crossover”) types of interdisciplinary research profile to evolve along multiple inter-dependent research pathways. Departure by the UK from the European Union presages major uncertainty about Science and Technology “framework funding” as represented by the EU’s Horizon 2020 research programming. This has meant a novel financing development bolstering research at Cambridge University has occurred as follows.

Because of UK (and EU) financial weakness, so-called “quantitative easing” more commonly known as “printing money” is practised by the Bank of England (and in the Eurozone, the European Central Bank). In the UK the Bank of England currently buys bonds issued by some universities, including Cambridge. The largest university bond was a £350 million issue from Cambridge in 2012 with a maturity date of 2052. Such bonds are sold to finance university research and teaching – deemed officially to make a material contribution to the UK economy. Accordingly, the Bank of England now also has a contributory role in funding long-term Cambridge University research (Wilson, 2016). As a final and recent indication of the financing prowess of the UK’s leading seats of academic entrepreneurship in the country’s changing circumstances, the following is indicative. A comparison of University venture funds shows the UK at the global top of the league (Table 1). Within the KAUST (King Abdullah University of Science & Technology of Saudi Arabia) University Venture Fund data for the UK, Cambridge Innovation Capital (a private fund) was a key investor in intellectual property, raising £75 million. From 2011 to 2016 University of Cambridge Enterprise (public knowledge transfer office of the university) administered deals involving 11 companies that were sold or stock exchange listed with a combined value of £1.3 billion. These spinouts own their own IP and were incubated in the university with regular peer-review of progress before coming to market. As hinted earlier, much of this initial investment capital comes from the Gulf and Asia (Frean, 2016)

**Table 1:** University venture funds

Country	Magnitude
UK	$5 billion
US	$4.5 billion
China	$2 billion
France	$1.1 billion
Japan	$0.6 billion

*Source*: KAUST Innovation Fund (2016)

.

Many of the implications of the global financial crisis, and some or all these listed key priorities, will as already noted, be affected by the UK exit from the EU with its negative and positive effects upon the cluster-platform. Thus access to high skilled migrant labour from the EU is directly affected by migration policy from the UK state. It is less a driver of negative effects than non-EU technological talent recruitment which, as we saw, is seen as often more modern in its curriculum than EU labour. Thus labour shortages may in different ways occur to EU and non-EU talent recruitment. As Segal et al. (2011) say, the cluster:

“...must recruit workers they need, recognising a particular shortage of top quality management and marketing skills but also the imperative to attract internationally excellent professionals from all spheres” (Segal et al., 2011, vi).

This means EU-start-ups, management and research leaders may continue to be sought while non-EU trained medical diagnosticians and analysts or technologists in medical and ICT fields will remain in demand. Finance will remain an imperative if high-tech growth occurs while the UK’s declining currency makes acquisitions from abroad more likely and attractive.

Finally, although Cambridge foreign acquisitions still occur as noted with Cambridge Consultants Ltd’s (CCL) acquisition of US firm Synapse, but such acquisitions became 20% dearer directly after the Brexit plebiscite (with assets 20% cheaper to outsiders). Until then this was part of CCL’s strategy to evolve a track record of creating high-value organisations built around disruptive technology, an exemplar of “thin” globalisation. Thus four of Cambridge’s $15 billion capitalisation firms - Cambridge Silicon Radio (CSR), Xaar, Vectura and Domino Printing Sciences - were among those spun off by CCL. Other spin-offs include Alphamosaic and Inca, who were subsequently acquired by Broadcom for $123 m and Japan’s Dainippon Screen for $60 m. With the expansion of its US presence, CCL would also be bringing its venturing activity to the extensively “financialized” US capital markets. Now there is greater uncertainty about basic and applied research funding that hitherto came to Cambridge research from programmes such as Horizon 2020. The UK government has given some reassurance that substitution of such funding will occur short-term, but the final arrangement awaits the results of Brexit negotiations. By contrast as shown, long-term uncertainty is in part insured against by the issuing of Cambridge University bonds that are currently available for purchase by the UK central bank’s quantitative easing policy, as long as it lasts. With inflation on the rise in 2017 monetary policy will remain constrained and interest rates straining but likely to be kept at historic lows during the UK’s “resilience-free” economic era, likely to last 10 years or more since the 2007–8 global financial crisis.

So, to return to South Korea’s current evolutionary arrest in its upward growth trajectory, South Korea is like an island, surrounded by sea and cut-off from the land-mass immediately to its north by an extremely hostile political power in North Korea, aided somewhat reluctantly in its political posture of local and global aggression by its globally prodigious geopolitical ally, China. Not surprisingly, South Korea, while globally competitive in consumer goods markets is quite locked-in to a manufacturing paradigm. It has become somewhat isolated regarding global good practice in industrial organization despite its success in the past in succeeding in rapid industrialisation. The Chaebol system by which industrial groups divide along sectoral boundaries, supported by an in-house domestic banking system, managed by an extremely hierarchical and rather bureaucratic administrative system, now seems rather outdated and lacking in forward impetus.

The problems of “cronyism” towards the disgraced former South Korean leader Park Geun-Hye with claims of enormous gifts to Presidential charitable interests and worse are testimony to the weakening of the traditional elite system of industry management. The scandal of the burning battery was one caused ultimately by a mentality that favoured “closed innovation” in a world that had generally become happier with market transactions involving “open innovation”. The in-house supplier chosen by Samsung was not expert in battery technology but SDI won the in-house supplier contract anyway. SDI stands for Samsung Digital Imaging which is actually an optoelectronics (or photonics) subsidiary of Samsung spun-off from Samsung Optoelectronics in 2009. The Burning battery in the Galaxy 7 smartphone cost Samsung some $5billion in 2016–7. Half the faulty batteries were produced by SDI while the other half were produced by affiliate Amperex Technology without fault. In a different field of Samsung operations where global networks with other major corporates are pronounced, Samsung Biologics is jointly owned by Samsung Electronics Co. and Samsung Everland Inc. each owning a 40% stake in the venture, with Samsung C&T Corp. and Durham, North Carolina-based Quintiles each holding 10%. Samsung Biologics will contract-make medicines comprising living cells. Samsung Group plans to expand into producing copies of biologics including Rituxan leukaemia and lymphoma treatments sold by Roche AG and Biogen Idec Inc. of Boston, MA. The Samsung Medical Centre is South Korea’s leading clinic. However, it transpired in 2015 that the national outbreak of a mutated form of Severe Acute Respiratory Syndrome (SARS) or (Middle Eastern IRS) originated in the Samsung Medical Centre. These, in different ways point to a worrying degree of “corporate overreach” by South Korea’s leading conglomerate.

## Conclusions

As a small-scale yet traditionally high-grade university research centre, Cambridge learnt the lesson of Intellectual Property in 1975 when local research launched a new industry – biotechnology – the commercial returns from which were exploited by academics and risk capitalists based across the world in the University of California’s San Francisco Medical School. Thereafter, a relatively liberal intellectual property regime prevailed with the discoverers or academic inventors evolving into innovators as they were encouraged to exploit their own IPR alongside that of the university by agreement. This meant that the growth trajectory self-guided towards an “open innovation” model of knowledge exploitation relatively unhindered by corporate requirements or interests except insofar as market-based contracts were fulfilled between global customers and local suppliers of commercialised knowledge-intensive output. Surrounding this research exploration/exploitation kernel, a facilitative bottom-up innovative and entrepreneurial infrastructure of “associative” intermediaries and “champions” evolved towards an economic governance form known in development studies as “Embedded Autonomy”. This is the opposite of a “Developmental State” model of economic growth once practised in early fast-growth Asian economies like South Korea, Singapore and Taiwan. As we saw earlier the last two of these, alongside Hong Kong, have largely moderated their “developmental state” models in favour of the “thin globalisation” of a knowledge-intensive “Quaternary” trajectory, deindustrialising away from manufacturing-led growth as they proceed. However, due to its deep path dependence on Chaebol-led state development thinking, South Korea has been unable or unwilling to join them, resulting in deteriorating economic indicators in consequence.

The self-sustaining ambition of he “Asian Tiger” pioneers has generally been to overcome the developmental blockage identified by Rodrik (2011), which has been to stimulate demand for innovation by stimulating the supply to meet demanding customers’ requirements for creating technology (not sector) entrepreneurs. Such technologies are then capable of becoming “general purpose technologies” not simply sector-limited technologies. Crossover innovations in leading Quaternary cluster-platform complexes like Silicon Valley, Cambridge and Israel naturally find applications among cluster ecosystems, especially where they co-exist in proximity with ready applications in new economic activities occurring outside their original technology base. Rather than relying upon corporate or state hierarchies to “pick winners” the local “Platform Champions” conduct local and national lobbying through their shared interest in promoting “Innovation Advocacy”. In the case of Cambridge anatomised above it began with the first champions for the nascent ICT cluster, and then evolved as a group of inter-related, albeit diverse clusters that subsequently grew into, currently, four pillars of a set of complementary, advanced technology knowledge-intensive or Quaternary cluster-platforms. From this, local-global marketing of “crossover” excellence leads to high employment, skills and profitability growth, something which all development experts hope to deliver.
